# Supplementary material for: Analysis of PPARγ Signaling Activity in Psoriasis
Source: Int J Mol Sci. 2021 Aug 10;22(16):8603. doi: 10.3390/ijms22168603 (PMC8395241; doi:10.3390/ijms22168603)
Supplement: Supplementary file 1 [file ijms-22-08603-s001.zip › Supplemental materials_Analysis of PPARg signaling activity in psoriasis/Pathway models/Models images and html files/Model of PPARG related pathways in psoriasis (short version)/42591.html]

muramyl dipeptide


# Small Molecule muramyl dipeptide

|  |  |
| --- | --- |
| URN | urn:agi-cas:53678-77-6 |
| Total Entities | 0 |
| Connectivity | 711 |
| Name | muramyl dipeptide |
| Molecular Weight | 492.477580 |
| XLogP | -3.700000 |

---

|  |  |
| --- | --- |
| Pathway | NOD-like Receptors |
|  | Toll-like Receptor Independent Sterile Inflammation |
|  | Macrophage Function in Diabetes Mellitus Type 1 |
|  | Crohn's Disease |
|  | Paneth-Cell Function in Crohn's Disease |
|  | Macrophage Dysfunction |
|  | Dendritic Cell Dysfunction in Crohn's disease |
|  | Dendritic Cell Function in Ulcerative Colitis |
|  | Blau Syndrome |
|  | Model of PPARG signaling in psoriasis |
|  | Model of PPARG related pathways in psoriasis (short version) |
|  | 1\_Insufficient activation of immune response in the middle ear epithelium cells in otitis media |
|  | 2\_Paneth cell dysfunction in Crohn's disease |
|  | 1\_Defects in response to pathogens in the gut promotes inflammation in Crohn's disease |
|  | 3\_Autophagy dysfunction in Crohn's disease |
|  | 2\_Intestine epithelial cells drive immunological responses in ulcerative colitis |

---

|  |  |
| --- | --- |
| MedScan ID | 1203017 |

---

|  |  |
| --- | --- |
| Alias | N(sup 2)-(N-(N-acetylmuramyl)-L-alanyl)-D-alpha-glutamine |
|  | EINECS 258-696-9 |
|  | N-acetylmuramyl-levo-alanyl-dextro-isoglutamine |
|  | BRN 4220745 |
|  | N-acetylmuramylalanyl-dextro-isoglutamine |
|  | N-acetylmuramyl-L-alanyl-D isoglutamine |
|  | N-Acetylmuramyl-L-alanyl-D-isoglutamine hydrate |
|  | 4-[2-[2-[3-acetylamino-2,5-dihydroxy-6-(hydroxymethyl)tetrahydropyran-4-yl]oxypropanoylamino]propanoylamino]-5-amino-5-oxo-pentanoic acid |
|  | 53678-77-6 |
|  | N-acetylmuramylalanyl-D-isoglutamine |
|  | N2-(N-(N-acetylmuramyl)-L-alanyl)-D-alpha-glutamine |
|  | Muramyl Dipeptide |
|  | N-acetyl muramyl-L-alanyl-D-isoglutamine |
|  | (+)-N2-(N-(N-acetylmuramyl)-L-alanyl)-D-alpha-Glutamine |
|  | 4-[2-[2-[3-acetylamino-2,5-dihydroxy-6-(hydroxymethyl)tetrahydropyran-4-yl]oxypropanoylamino]propanoylamino]-5-amino-5-oxo-pentanoate |
|  | ac-muramyl-ala-d-glu-nh2 |
|  | N-(N-acetylmuramyl)-L-alanyl-D-alpha-Glutamine |
|  | Acetylmuramyl-alanyl-isoglutamine |
|  | N2-(N-(N-Acetylmuramoyl)-L-alanyl)-D-alpha-glutamine |
|  | (+)-N2-(N-(N-acetylmuramoyl)-L-alanyl)-D-alpha-Glutamine |
|  | N-(N-acetylmuramoyl)-L-alanyl-D-alpha-Glutamine |
|  | N(sup 2)-(N-(N-Acetylmuramoyl)-L-alanyl)-D-alpha-glutamine |
|  | D-alpha-Glutamine, N-(N-acetylmuramoyl)-L-alanyl- |
|  | Adjuvant Peptide |
|  | D-alpha-Glutamine, N2-(N-(N-acetylmuramoyl)-L-alanyl)- |
|  | D-alpha-Glutamine, N2-(N-(N-acetylmuramoyl)-L-alanyl)-, (+)- |
|  | D-alpha-Glutamine, N(sup 2)-(N-(N-acetylmuramoyl)-L-alanyl)- |

---

|  |  |
| --- | --- |
| CAS ID | 53678-77-6 |

---

|  |  |
| --- | --- |
| Reaxys ID | 4220745 |

---

|  |  |
| --- | --- |
| InChIKey | MEJOVVKHFORGKW-HOMBLFBHSA-N |
|  | BSOQXXWZTUDTEL-IXJLSLDXSA-N |

---

|  |  |
| --- | --- |
| Molecular Formula | C19H32N4O11 |

---

|  |  |
| --- | --- |
| PubChem SID | 135004313 |
|  | 181651 |

---

|  |  |
| --- | --- |
| PubChem CID | 91667728 |
|  | 40846 |

---

|  |  |
| --- | --- |
| XLogP-AA | -4.6 |

---

|  |  |
| --- | --- |
| IUPAC Name | (4R)-4-[[(2S)-2-[[(2R)-2-[(1R,2R,3R)-1-[(1R)-1-acetamido-2-keto-ethyl]-2,3,4-trihydroxy-butoxy]propanoyl]amino]propanoyl]amino]-5-amino-5-keto-valeric acid |
|  | (4R)-4-[[(2S)-2-[[(2R)-2-[(2R,5S)-3-acetamido-2,5-dihydroxy-6-methylol-tetrahydropyran-4-yl]oxypropanoyl]amino]propanoyl]amino]-5-amino-5-keto-valeric acid |

---

|  |  |
| --- | --- |
| Rotatable Bond Count | 16 |
|  | 12 |

---
